# Surface engineering enables robust SEI growth towards a stable and efficient lithium-ion battery SiO_*x*_ anode

**DOI:** 10.1039/d5ra08407e

**Published:** 2026-01-07

**Authors:** Fan Wu, Hongcao Shi, Guijia Hu, Xiangshun Yan, Zihao Wang, Jinhao Shu, Gaoyuan Chen, Yongshu Wang, Jianwei Wang, Yuan Chen

**Affiliations:** a College of Mechanical and Electronic Engineering, Shandong University of Science and Technology Qingdao 266590 China chenyuan@sdust.edu.cn; b College of Energy Storage Technology, Shandong University of Science and Technology Qingdao 266590 China; c Key Laboratory of Intelligent Optoelectronic Devices and Chips of Jiangsu Higher Education Institutions, School of Physical Science and Technology, Suzhou University of Science and Technology Suzhou 215009 China; d Advanced Technology Research Institute of Taihu Photon Center, School of Physical Science and Technology, Suzhou University of Science and Technology Suzhou 215009 China

## Abstract

It is challenging to enhance the cycling stability and rate capability of SiO_*x*_ (0 < *x* < 2) anodes for commercialization, due to the random and disordered growth of the solid electrolyte interphase (SEI) on the anode surface. Here, a surface engineering strategy was proposed to controllably regulate SEI formation. Through *in situ* constructing and densely coating a C–N network on the SiO_*x*_ nanoparticle surface, the surface energy and electronic structure were regulated, resulting in controlled growth of SEI components. The optimized SEI architecturally consists of inner Li_2_O and an outer LiF/Li_2_CO_3_ mixture. It not only enables mechanical robustness but also suppresses electrolyte decomposition, which significantly improves the Li^+^ transport kinetics at the electrode/electrolyte interfaces, resulting in a 4-fold reduced interfacial charge transfer resistance. Consequently, the anode exhibits outstanding electrochemical performance, with an initial reversible capacity of 1674 mAh g^−1^. Moreover, the high capacities of 1618 mAh g^−1^, 1274 mAh g^−1^ and 1114 mAh g^−1^ were recorded at the 100th, 200th, and 300th cycles with a 1 A g^−1^ current density, respectively. The capacity retention rates were 96%, 83%, and 66%, respectively, which demonstrates good cycling stability. Besides, the rate capability approached 888 mAh g^−1^ at 5 A g^−1^ and recovered 98% as the current decreased to 0.1 A g^−1^. More importantly, this method is low-cost, scalable, and uniform, making it suitable for large-scale industrial applications. This work provides a new way of producing high-performance SiO_*x*_ anodes; moreover, the scalable fabrication is promising for industrial applications.

## Abstract 2

It is challenging to enhance the cycling stability and rate capability of SiO_*x*_ (0 < *x* < 2) anodes for commercialization, due to the random and disordered growth of the solid electrolyte interphase (SEI) on the anode surface.

## Graphical abstract

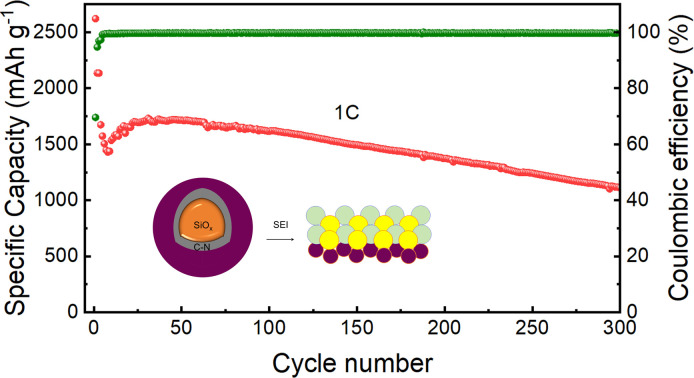

## 1 Introduction

The demand for high-energy-density lithium-ion batteries (LIBs) is rising with the development of electric vehicles and mobile electronic devices, but traditional graphite anodes cannot meet the increasing requirements, driving the need for research into high-capacity anodes.^1–4^ SiO_*x*_ with medium volume expansion (∼113%) and outstanding reversible capacity (∼2000 mAh g^−1^) has been looked at as an ideal candidate.^5–7^ However, in actual applications, it is still limited by a low reversible capacity, short cycle lifetime, and poor rate performance.^8^ All of these limitations have been demonstrated to derive from the solid electrolyte interphase, which forms on the SiO_*x*_ anode surface during the initial charge–discharge stage.^9^ As reported, the SEI is constructed from organic and inorganic layers. The organic layer is composed of electrolytes and provides good elasticity and ion-free transport channels. Conversely, the inorganic layer consists of Li_2_CO_3_, Li_2_O, and LiF, respectively. Among them, LiF presents a high Young's modulus, which enables good mechanical properties, and Li_2_CO_3_ exhibits good electron insulation, which can effectively suppress redox reactions. Li_2_O shows poor electronic/ionic conduction and usually serves as a filler.^10^ Their stoichiometry and spatial distribution not only affect the mechanical strength but also determine the ionic/electron diffusion/transport rate.^11,12^ Thus, constructing an SEI with reliable mechanical performance, high ion conductivity, and electronic insulation is the key to obtaining ideal electrochemical performance.^13–15^

Currently, techniques such as anode surface engineering, artificial SEI, electrolyte design, and electrodeposition have been reported to regulate the SEI.^16–21^ However, major bottlenecks persist, including: (1) complex, costly, and low-yield SEI manufacturing; (2) imprecise control over the molecular constituents and structure of the SEI; and (3) electrochemical and mechanical incompatibility with battery components. Among them, surface engineering, applied during the synthesis stage, is an effective strategy. It is carried out through coating, doping, and etching to regulate the surface atomic arrangement, electronic structure, surface energy, *etc.*, achieving controllable SEI composition, crystallinity, and spatial distribution.^22^ Carbon coating is the most common route in surface engineering. It not only changes the surface properties but also relieves the volume expansion of SiO_*x*_. Common methods include ball milling, chemical vapor deposition, sol–gel, and hydrothermal synthesis. Moreover, introducing heteroatoms into the carbon layer can further regulate its conductivity, surface energy, active sites, and electrochemical properties.^23^ The reported heteroatoms include non-metal elements (B, N, P, S, F),^24–28^ and metal elements (Mg, Co, Fe, Mo, Ti, Ag).^29–34^ Although the positive effect of heteroatom-doping has been widely demonstrated, it was still limited from the perspective of controllable composition homogeneity, carbon layer thickness and densification. More importantly, its effect on SEI growth should be further clarified. Therefore, addressing these aspects is essential for accurately constructing a core–shell structure and ultimately enhancing the electrochemical performance of SiO_*x*_ is necessary.

To surmount the bottleneck of unregulated SEI formation limiting SiO_*x*_ anode commercialization, a dedicated surface engineering strategy is presented for controllable SEI modulation. This study establishes a new avenue for fabricating high-performance SiO_*x*_ anodes, laying a solid foundation for their large-scale industrial deployment.

## 2 Experimental section

### 2.1 The synthesis of the anode material

The anode composite material with 10 wt% N was prepared by using the solid-state reaction method. First, commercial SiO_*x*_ powder (purity ≥ 99%, particle size 100 mesh), melamine (C_3_H_6_N_6_, AR) and glucose (C_6_H_12_O_6_, purity ≥ 99.5%) were mixed with a weight ratio of 6 : 2 : 2 and milled for 4 hours (zirconia balls, ethanol, 180 rpm). Then, the as-obtained powder was heated at 200 °C for 2 h in a vacuum drying oven. After that, the powder was milled for 1 h. Subsequently, the powder was sealed in a high-temperature furnace, heated at a rate of 5 °C min^−1^ to 900 °C under a N_2_ atmosphere (flow rate 50 sccm), and calcined for 1 h. Finally, the anode material was produced.

### 2.2 Material characterization

Scanning electron microscopy (SEM) images were recorded by a TESCAN MIRALMS, and transmission electron microscopy (TEM) and high-resolution TEM images were collected with a JEOL JEM-2100F TEM. X-ray photoelectron spectroscopy (XPS) analysis was carried out on a K-Alpha. Fourier-transform infrared (FT-IR) measurements were performed on a Nicolet iS10 Fourier-transform infrared spectrometer. Inductively coupled plasma (ICP) measurements were carried out on a Thermo Scientific iCAP 7400 ICP-OES.

### 2.3 Coin cell assembly

The CN–SiO_*x*_, Super P, and sodium alginate were mixed with a weight ratio of 7 : 2 : 1. The electrode slurry was prepared using deionized water as the solvent and coated on Cu foil. The loading was controlled from 0.8 to 1.2 mg cm^−2^. The as-obtained electrodes were dried in a vacuum oven at 60 °C for 12 h, and the electrodes were punched into discs with a diameter of 12 mm. The polished Li foil was punched into 14 mm diameter, and 200 µm thick discs. CR2032 coin cells were assembled in an argon-filled glove box, using a lithium disc as the counter electrode and a PP separator with 80 µL of electrolyte. After that, the batteries were rested at 25 °C for 12 h.

### 2.4 Electrochemical measurements

A CT-4008T-5V10mA-164 battery testing system was used to measure the galvanostatic charge/discharge of the coin cells. Cyclic voltammetry (CV), electrochemical impedance spectroscopy (EIS), and Galvanostatic Intermittent Titration Technique (GITT) measurements were carried out on a CHI660E electrochemical workstation (Shanghai Chenhua Instrument Co., Ltd).

### 2.5 Theoretical calculations

All calculations were carried out using density functional theory (DFT) within the Vienna *Ab initio* Simulation Package (VASP). The electron exchange–correlation functional was treated using the Perdew–Burke–Ernzerhof generalized gradient approximation (PBE-GGA), and the ion–electron interactions were described by the projector augmented wave (PAW) method. Monkhorst–Pack *k*-point meshes with a spacing of 0.25 Å^−1^ were used for the N-doped graphite/graphite/SiO_*x*_@SEI slab models. Geometry optimization was performed until the total energies (atomic forces) converged to 10^−4^ eV (0.01 eV Å^−1^) with a plane-wave energy cutoff of 400 eV.

## 3 Results and discussion

**Fig. 1:**
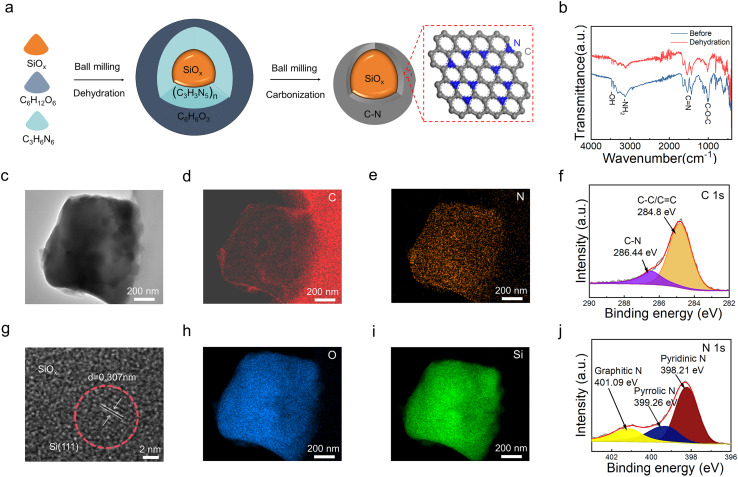
(a) Schematic illustration of the synthesis and structural features. (b) Fourier transform infrared spectroscopy before and after dehydration condensation treatment. (c) HAADF-STEM images of CNS. (d–i) Elemental mapping images of C, Si, N, and O. (g) HRTEM images of CNS. (f and j) High-resolution XPS spectra of C 1s and N 1s.

Fig. 1a displays the preparation process of the SiO_*x*_ anode with an N-doped carbon network and coating (named as CNS). Briefly, glucose (C_6_H_12_O_6_) and melamine (C_3_H_6_N_6_) were used as carbon and nitrogen sources, respectively. They were uniformly mixed with SiO_*x*_ powder and ball-milled to form a preliminary coating, followed by vacuum drying to remove their internal water molecules (dehydration reactions). During this process, preliminary C–N network formation occurred through the condensation of melamine and glucose. Subsequently, the mixture was further ball-milled to reduce the particle size and achieve a uniform coating of the carbon precursor. After that, it was calcined under an argon atmosphere for several hours. Finally, the CNS was produced; as a comparison, carbon-coated SiO_*x*_ (named as CS) was prepared using the same procedure but without nitrogen source addition. The microstructure was characterized by TEM. Fig. 1c shows that the CNS particles are below one micrometer in size. The high-resolution TEM showed partial lattice fringes (Fig. 1g), corresponding to the Si (111) plane, indicating that partially crystalline silicon exists within it. Fig. 1d, e, h and i display the elemental mapping images of C, N, O, and Si in the CNS, respectively. This not only confirms that the C layer is fully and uniformly coated onto the SiO_*x*_ particles but also shows that the N element is uniformly dispersed on the C layer. This suggests that the *in situ* dehydration process at the particle scale was the key to forming a dense and uniform coating. The removal of –OH and –NH_2_ groups leaves behind ether bonds (–C–O–C–) and C=N groups (Fig. 1b). The formed polymer and C=N bonds enable complete encapsulation of the SiO_*x*_ particles and facilitate the incorporation of N atoms into the carbon framework, preventing their escape at high temperatures.

Then, X-ray photoelectron spectroscopy (XPS) was used to investigate the elemental valence states. The C 1s spectrum was fitted with two characteristic peaks located at 284.8 eV and 286.44 eV (Fig. 1f), corresponding to C–C and C–N bonds.^35–37^ This result indicates that the carbon in the composite material exists in sp^2^ and sp^3^ hybridization states, along with residual functional groups, and the N 1s spectrum shown in Fig. 1j has three peaks at 398.21 eV, 399.26 eV, and 401.09 eV, which can be attributed to pyridine-N, pyrrole-N, and graphitic-N, respectively.^38^ These results suggest that N-doping has been introduced into the carbon layer. These two peaks (284.8 eV and 286.44 eV) correspond to the amorphous and graphite carbon, respectively. The Si 2p spectrum (Fig. S1) displays characteristic peaks that appear at 99.2 eV (Si^0^) and 102.9 eV (Si^2+^), further evidencing the presence of crystalline silicon. Furthermore, structural, morphological, and compositional evaluations of the as-prepared samples from large-scale batches confirm their good consistency (Fig. S2).

**Fig. 2:**
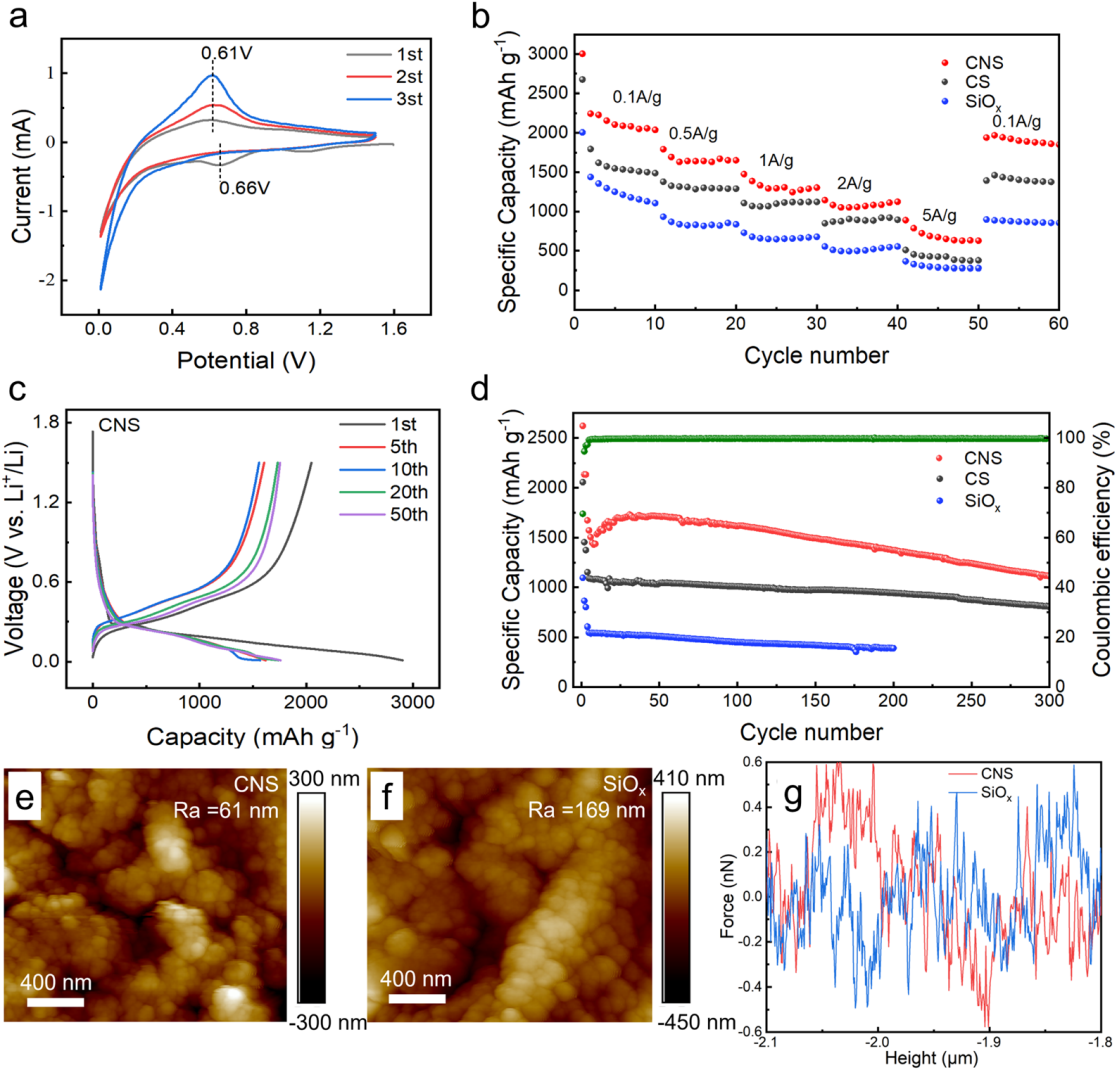
(a) CV profiles of CNS at 0.1 mV s^−1^. (b) Rate performance. (c) Discharge–charge voltage curves of CNS at 100 mAh g^−1^. (d) Cycling performance at 1.0 A g^−1^. (e) AFM height map of the CNS electrode. (f) AFM height map of the SiO_*x*_ electrode. (g) Force–distance curves of CNS and SiO_*x*_.

Fig. 2a shows the cyclic voltammetry (CV) curve of CNS at 0.1 mV s^−1^, and it can be seen that the oxidation and reduction peaks with good symmetry appear at around 0.61 V and 0.66 V, respectively. Compared to the CS, which presents two oxidation peaks at 0.38 V/0.54 V and one reduction peak at 0.15 V (Fig. S3), the sharper peaks of CNS indicate easier redox reactions and higher electrochemical activity. The higher response current corresponds to improved reaction kinetics. Moreover, the smaller potential difference between the oxidation and reduction peaks suggests better stability and reversibility of the SEI.^39^Fig. 2b shows the rate capability at different current densities ranging from 0.1 to 5 A g^−1^. As shown, the CNS has a discharge capacity of 2000 mAh g^−1^, 1600 mAh g^−1^, 1200 mAh g^−1^, and 888 mAh g^−1^ at the current densities of 0.1, 0.5, 1, 2, and 5 A g^−1^, respectively. Moreover, as the current densities recover to 0.1, the discharge capacity is 1900 mAh g^−1^, retaining 98% of its initial value. In comparison, the CS and SiO_*x*_ show poor discharge capacity and recovery rate, indicating that the CNS has higher capacity and rate performance. Additionally, the charge/discharge curve of CNS is shown in Fig. 2c, and presents a high initial reversible capacity approaching 3000 mAh g^−1^. Although the lower initial coulombic efficiency (∼70%) was significantly reduced, the capacity of 1680 mAh g^−1^ at the 5th cycle was recorded. Moreover, it shows good coincidence at the 10th, 20th, and 50th cycles, indicating the excellent cycling stability of CNS. In contrast, the CS shows a smaller initial reverse capacity (∼2000 mAh g^−1^), initial coulombic efficiency (∼67%), and steady-state capacity (∼1230 mAh g^−1^) (Fig. S4). Similarly, the SiO_*x*_ shows poor initial reverse capacity (∼1700 mAh g^−1^), initial coulombic efficiency (∼62%), and capacity (∼900 mAh g^−1^) (Fig. S5). Both carbon and N-doped carbon coating on the SiO_*x*_ particles have significantly increased the cycling stability and reverse capacity, which is in agreement with the results reported above. This is because carbon coating on SiO_*x*_ buffers expansion, stabilizes the SEI and enables electron transport, while nitrogen doping boosts the active sites for Li^+^ intercalation and conductivity. Their synergy enhances the cycling stability and reversible capacity.^40^Fig. 2d shows the cycling performance of CNS, CS, and SiO_*x*_. The CNS retains a high specific capacity and smooth charge/discharge cycles within 300 times. The specific capacity was 1600 mAh g^−1^, 1400 mAh g^−1^, and 1140 mAh g^−1^ at the 100th, 200th, and 300th cycles, respectively. The capacity was 96%, 84%, and 67%, respectively. Moreover, the coulombic efficiency was maintained over 99%, indicating good stability. In comparison, the CS also shows good stability and maintains about 70% capacity after 300 cycles, but a lower specific capacity (1000 mAh g^−1^). Meanwhile, the SiO_*x*_ shows both poor specific capacity (600 mAh g^−1^) and stability.

Besides, the characteristic capacity curve of the CNS electrode shows a notable recovery capacity around the initial 20 cycles, which is intricately linked to the dielectrophoresis effect that is derived from the C–N coating layer.^41^ The dielectrophoresis effect could be described briefly as follows: as the current during the charging process increases from 0.1C to 1C, the electrode rapidly expands, causing some of the alloy phase Li_*x*_Si to detach from the copper foil surface and enter the electrolyte, forming a neutral state. Subsequently, the irregular electric field causes it to move back to the electrode surface and re-work from the neutral state, resulting in capacity recovery. N-doping enables the good conductivity of CNS, which results in the generation of localized non-uniform electric fields during the charge–discharge stage. Meanwhile, the SiO_*x*_ and CS did not exhibit capacity fluctuation, which could contribute to their poor conductivity and inability to generate sufficient irregular electric fields. As shown in Fig. 2e and f, the surface roughness of CNS was ∼61 nm, while the surface roughness of SiO_*x*_ was 169 nm. This indicates that the CNS morphology is more uniform. The force–height curve (Fig. 2g) suggests that the CNS has a higher slope between the force maximum and zero points, which indicates higher hardness and elastic modulus, and is higher than that of SiO_*x*_.^42^

**Fig. 3:**
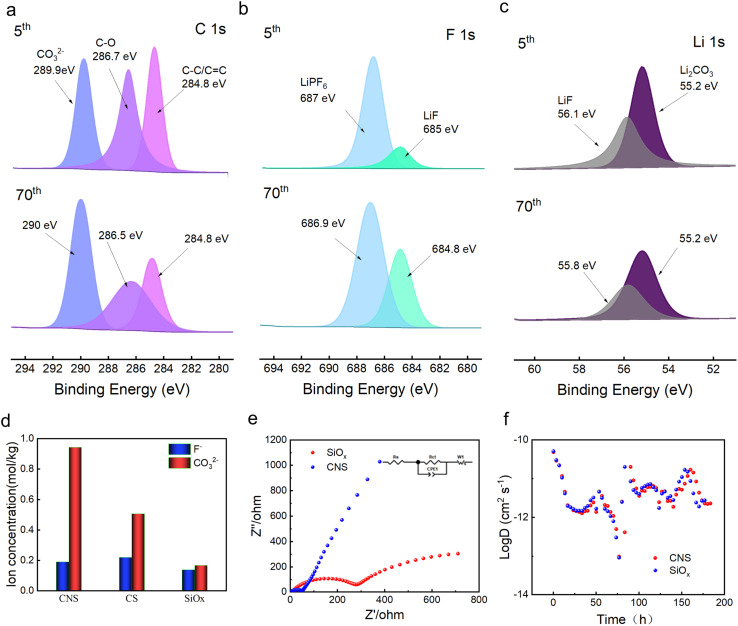
(a–c) Comparison of the high-resolution XPS spectra of C 1_S_, F 1_S_, and Li 1_s_ after the 5th and 70th cycles of CNS. (d) Comparison of the F^−^ and CO_3_^2−^ concentrations at the first activation of SiO_*x*_, CS, and CNS. (e) EIS comparison between CNS and CS before cycling. (f) Comparison of the GITT profiles and calculated D of CNS and SiO_*x*_.

The CNS shows superior electrochemical performance compared to both SiO_*x*_ and CS. To investigate the mechanism, further characterizations were conducted. First, the electrodes after the 5th and 70th cycles were disassembled. Then, the surface elemental valence states were tested using XPS. The C 1s spectrum after the 5th cycle could be fitted to three peaks located at 284.8 eV, 286.7 eV, and 289.9 eV, respectively (Fig. 3a). They were assigned to C–C/C=C, C–O, and CO_3_^2−^, respectively, where the C–C/C=C and C–O were derived from the decomposition products of the organic electrolyte, and the CO_3_^2−^ was attributed to the Li_2_CO_3_ of the SEI inorganic layer. In comparison, the CO_3_^2−^ peak after the 70th cycle has increased 0.1 eV. The two fitted F 1s spectra after the 5th cycle were observed at 687 eV and 685 eV (Fig. 3b), which could be attributed to LiPF_6_ and LiF, respectively. After the 70th cycle, they decreased by 0.2 eV. The two Li 1s peaks after the 5th cycle were located at 56.1 eV and 55.2 eV (Fig. 3c), which could be attributed to LiF and Li_2_CO_3_, respectively. Moreover, after the 70th cycle, they decreased by 0.3 eV. The shifted element binding energies for C 1s, F 1s and Li 1s suggest that LiF and Li_2_CO_3_ gradually form a robust and heterogeneous interface during cycling, enhancing electron transfer between the two phases.^43^ It also indicates that the composition and spatial distribution of the SEI are not constant, but dynamically evolve during the cycling process.^44^ However, the Li 1s peaks of SiO_*x*_ show three peaks corresponding to Li_2_O, Li_2_CO_3_ and LiF, respectively (Fig. S6). This indicates that the N-doped carbon coating in CNS effectively suppresses the formation of Li_2_O.^45^

As reported, Li_2_O was a byproduct derived from the reaction between the electrolyte and impurities (such as EC, and residual SiO_*x*_), which is inconducive to Li^+^ transport and mechanical properties. Regarding the CS electrode, the Li_2_O is not detected after the 5th cycle, but it was found at the 70th cycle (Fig. S7), which suggests that the reduction process causes Li_2_O to be continuously generated, while the outer layer of Li_2_CO_3_ and LiF becomes loose and partially consumed. To accurately obtain the content of carbonate and fluoride in the SEI, ion chromatography was performed. The SEI layer was washed with KOH and methanesulfonic acid to remove the residual surface organic electrolyte, and to prepare the test sample, it was volumetrically made up to volume with pure water, shaken and sonicated for 1 h, and finally diluted with pure water. As shown in Fig. 3d, for CO_3_^2−^, CNS > CS > SiO_*x*_, while for F^−^, SiO_*x*_ < CNS < CS. This indicates that the Li_2_CO_3_ in the SEI was the critical component for electrochemical performance, due to its moderate ionic conductivity and good chemical stability. To investigate the charge transfer capability of the SEI, Fig. 3e displays the Nyquist plots of the CNS and CS electrodes. The *R*_ct_ value was fitted with the equivalent circuit shown in Fig. 3e inset, and it shows a value of 60.56 Ω and 240 Ω for CNS and SiO_*x*_, respectively. The N-doping obtained a quarter of the charge transfer resistance, which indicates significantly improved conductivity.^46^ Furthermore, the Galvanostatic Intermittent Titration Technique (GITT) was employed to evaluate the Li^+^ transport kinetics of the SEI, and the diffusion coefficient (*D*) was calculated using eqn (1).^47,48^1
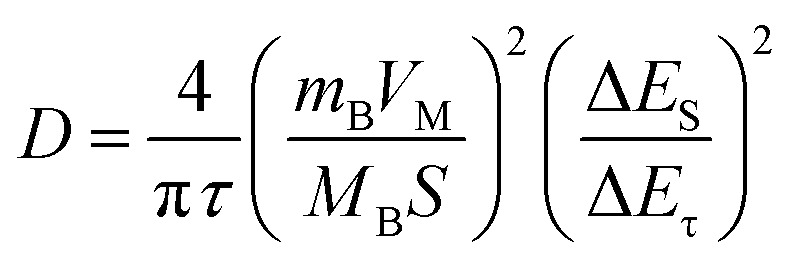


**Fig. 4:**
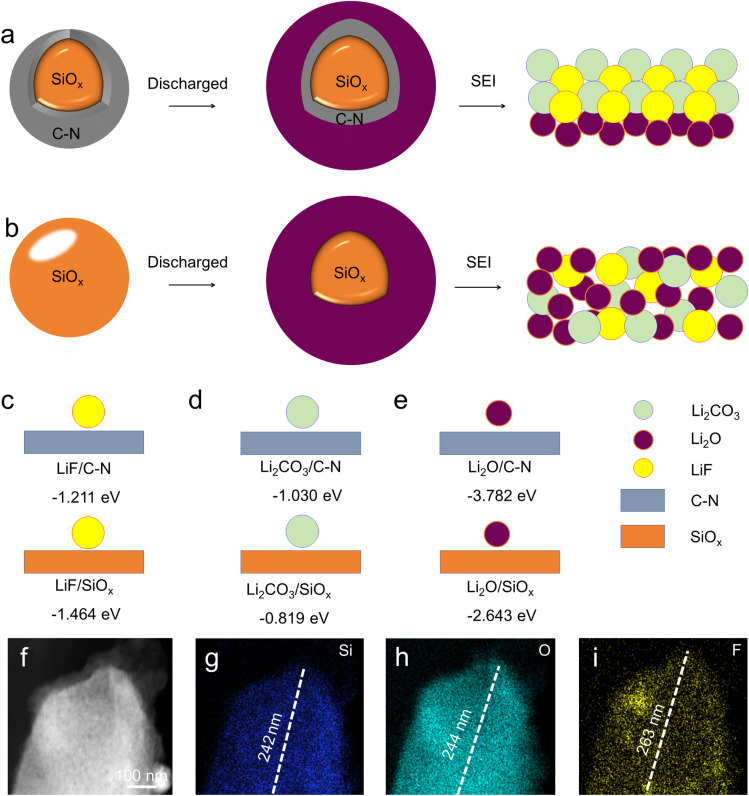
(a) Schematic diagram of the interface of CNS during discharge. (b) Schematic diagram of the interface of SiO_*x*_ during discharge. (c–e) Schematic diagram of the energy values corresponding to LiF/Li_2_CO_3_/Li_2_O at the interface of CNS and SiO_*x*_. (f) HAADF-STEM image of a CNS sample. (g–i) Elemental mapping images of Si, O, and F.

Fig. 3f shows the diffusion coefficient of CNS and CS with logarithm value. It indicates that the value for the CNS is higher than that for the CS. This indicates that the SEI of the CNS has a better ion diffusion ability. Based on the above results and discussion, we conclude that the CNS significantly improves the reversible capacity, rate performance, and long-term cycling stability, owing to the formation of a well-compacted LiF/Li_2_CO_3_ layer on the same plane. This not only enables higher electron conductivity but also maintains a higher ion diffusion coefficient (Fig. 4a). Meanwhile, for the SiO_*x*_ alone, the SEI presents a loose distribution, which readily facilitates the growth of lithium dendrites, resulting in poor electron conductivity and ion diffusion coefficient (Fig. 4b). During cycling, the SEI was easily damaged due to the electrode volume expansion, causing rapid growth of lithium dendrites.^49^ Thus, the reversible capacity, rate performance, and long-term cycling stability deteriorate.

To further understand the components and spatial distribution formation of the SEI during the activation stage, density functional theory (DFT) calculations were performed. The adsorption energies of LiF/Li_2_CO_3_/Li_2_O on N-doped graphite, graphite, and SiO_*x*_ were calculated, respectively. The smaller absorption of energy corresponds to easier formation. As a result, the adsorption energy was −3.78 eV, −1.21 eV, and −1.03 eV for CNS. This indicates that Li_2_O was more likely to form in the inner layer of the SEI, while LiF and Li_2_CO_3_ tend to form a dense and compact outer layer, effectively sealing the electrode surface, in agreement with the XPS results. Meanwhile, for SiO_*x*_, the adsorption energies of Li_2_O, LiF, and Li_2_CO_3_ were −2.64 eV, −1.46 eV, and −0.81 eV, respectively, which shows an obvious gradient energy distribution and may cause a loose arrangement of the three components (Fig. 4c and d). Moreover, for CS, the adsorption energies of Li_2_O, LiF, and Li_2_CO_3_ were −0.025 eV, −0.027 eV, and −0.04 eV, respectively (Fig. S8). The nearly identical adsorption energies indicate random and disordered growth. All these results indicate that the surface composition and spatial distribution can be controlled. After that, we performed the TEM element mapping of the CNS after SEI formation (Fig. 4f–i), and the distribution ranges for Si, O, and F were 242 nm, 244 nm, and 263 nm, respectively. The smaller range of O than F indicates that the Li_2_O was distributed in the inner layer, and LiF was located in the outer layer. Based on the results above, we conclude that the SEI structure is Li_2_O/LiF@Li_2_CO_3_.

**Fig. 5:**
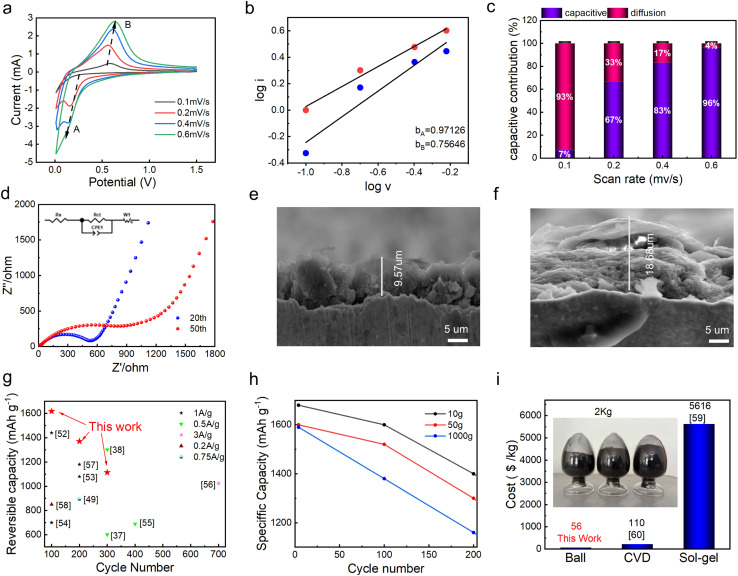
(a) CV curves of CNS at different scan rates. (b) Log *i vs.* Log *v* plots for electrode kinetics analysis. (c) Capacitive and diffusion contributions to charge storage at various scan rates. (d) EIS comparison of CNS after 20 and 50 cycles. (e) Cross-sectional SEM image of the CS electrode after cycling. (f) Cross-sectional SEM image of the CNS electrode after cycling. (g) Capacity retention rate of this work compared with previous studies. (h) Specific capacity of CNS at different preparation scales (10, 50, and 1000 g) during cycling. (i) Production cost per kilogram for samples prepared by ball milling, CVD, and sol–gel methods.

The reaction kinetics were investigated by cyclic voltammetry (CV) at scan rates ranging from 0.1 to 0.6 mV s^−1^ within a potential window of 0.01–1.5 V (Fig. 5a). The redox peaks were observed at ∼0.6 V and ∼0.2 V, corresponding to the lithium insertion/extraction in the anode. Moreover, as the scan rate rises, the peak current and potential difference increase, indicating that the process is dominant from electrode polarization, and the repeatable curve indicates good rate response ability. Furthermore, the CV curves were fitted with log *i*–log *v* (Fig. 5b), and the relationship between peak current and scan rate can be described with eqn (2)–(4):^50^

2 *i* = *av*^*b*^

3 log *i* = log *a* + *b* log *v*

4 *i*(*v*)=*k*_1_*v* + *k*_2_*v*^1/2^

The obtained slopes (*b*) for the reduction and oxidation peaks were *b*_A_ = 0.97126 and *b*_B_ = 0.75646, respectively. A *b*-value close to 1 indicates a capacitively controlled electrode process. This means that lithium ions could rapidly move on the anode surface, which results in high rate and fast-charging performance of the LIBs. Fig. 5c shows the contribution ratios of the capacitive and diffusion-controlled processes at different scan rates. As the scan rate increases, the capacitive percentage increases from 7% to 96%, confirming that lithium-ion storage becomes dominated by surface-controlled mechanisms at high rates.^46^ This endows fast charging, which helps to mitigate the rate limitations typical of silicon-based materials.^51^Fig. 5d displays the Nyquist plots of CNS electrode after the 20th and 50th cycles. The charge transfer resistance (*R*_ct_) was obtained by fitting the data with the equivalent circuit shown in the inset of Fig. 5d, yielding values of 532 Ω and 806 Ω after the 20th and 50th cycles, respectively. Moreover, the *R*_ct_ and the impedance related to the solid-state diffusion process (reflected by the Warburg impedance and low-frequency region) indicate that the CNS anode maintains good structural integrity and interface stability, with no severe side reactions at the electrode/electrolyte interface. Fig. 5e and f show the cross-sectional SEM images of the CNS and SiO_*x*_ electrode after cycling. The CNS electrode has a smaller thickness of 9.57 µm, maintaining a compact structure. This indicates that volume expansion is effectively suppressed. The SiO_*x*_ electrode presents a thickness of 18.68 µm, exhibiting severe structural agglomeration and volume expansion. Fig. 5g shows the statistics and comparison of the reversible capacity at different cycling times with the literature.^37,38,52–58^ The CNS presents competitive reversible capacities and stability of ∼1600 mAh g^−1^ at 100 cycles, ∼1400 mAh g^−1^ at 200 cycles, and ∼1100 mAh g^−1^ at 300 cycles, respectively, demonstrating the superiority of the surface structure design method to regulate SEI performance. Beyond cycling stability and capacity retention, practical industrial application requires consideration of scalability, cost, and production efficiency. Here, for further description, Fig. 5h is given to illustrate the scalability advantages, showing the specific capacity of CNS samples prepared at 10 g, 50 g, and 1000 g scales. After 4 cycles, their specific capacity is 1680 mAh g^−1^, 1600 mAh g^−1^, and 1590 mAh g^−1^, respectively. In addition, the sample prepared at the scale of 1000 g at 200 cycles still maintains a specific capacity of 1160 mAh g^−1^. This proves that the capacity decay of the materials is within a reasonable and acceptable range during mass production and has the potential to adapt to industrial mass production. Fig. 5i shows the expense per kilogram of CNS compared with that of CVD and sol–gel methods.^59,60^ The inset in Fig. 5i shows a photograph of the CNS samples, with costs of 56$, 110$ and 5616$ per kilogram respectively. This implies that the CNS has a cost advantage in terms of expense per kilogram, indicating that the preparation strategy of CNS may be conducive to large-scale application.

## 4 Conclusions

In conclusion, we have reported a surface engineering strategy for controllable growth of the SEI on the SiO_*x*_ anode. Through *in situ* constructing and densely coating a C–N network on the SiO_*x*_ nanoparticle surface, the surface energy and electronic structure were regulated, resulting in controlled growth of SEI components. The optimized SEI architecture features an inner layer of Li_2_O and an outer mixture of LiF and Li_2_CO_3_. It not only enables mechanical robustness but also suppresses the electrolyte decomposition, which significantly improves the Li^+^ transport kinetics at the electrode/electrolyte interfaces, resulting in a 4-fold reduced interfacial charge transfer resistance. Consequently, the anode exhibits outstanding electrochemical performance, and this method is low-cost, scalable, and uniform, making it suitable for industrial applications.

## Conflicts of interest

There are no conflicts to declare.

## Supplementary Material

RA-016-D5RA08407E-s001

## Data Availability

The data that support the findings of this study are available from the corresponding author upon reasonable request. Supplementary information (SI) is available. See DOI: https://doi.org/10.1039/d5ra08407e.
