# Supplementary material for: Surface engineering enables robust SEI growth towards a stable and efficient lithium-ion battery SiOx anode
Source: RSC Adv. 2026 Jan 7;16(2):1977–87. doi: 10.1039/d5ra08407e (PMC12775912; doi:10.1039/d5ra08407e)
Supplement: RA-016-D5RA08407E-s001 [file RA-016-D5RA08407E-s001.pdf]

Supplementary Information (SI) for RSC Advances.
This journal is © The Royal Society of Chemistry 2025

Surface Engineering Enables Robust SEI Growth towards Stable and Efficient Lithium-Ion Batteries SiO_x Anode

Fan Wu,[1,2] Hongcao Shi,[1] Guijia Hu,[2] Xiangshun Yan,[1] Zihao Wang,[1] Jinhao Shu,[1] Gaoyuan Chen,[3,4] Yongshu Wang,[2] Jianwei Wang,[2] Yuan Chen[1]*

[1]*College of Mechanical and Electronic Engineering, Shandong University of Science and Technology, Qingdao 266590, China.*

[2]*College of Energy Storage Technology, Shandong University of Science and Technology, Qingdao 266590, China.*

[3]*Key Laboratory of Intelligent Optoelectronic Devices and Chips of Jiangsu Higher Education Institutions, School of Physical Science and Technology, Suzhou University of Science and Technology, Suzhou, 215009, China*

[4]*Advanced Technology Research Institute of Taihu Photon Center, School of Physical Science and Technology, Suzhou University of Science and Technology, Suzhou, 215009, China*

*Corresponding author

E-mail: *chenyuan@sdust.edu.cn*.

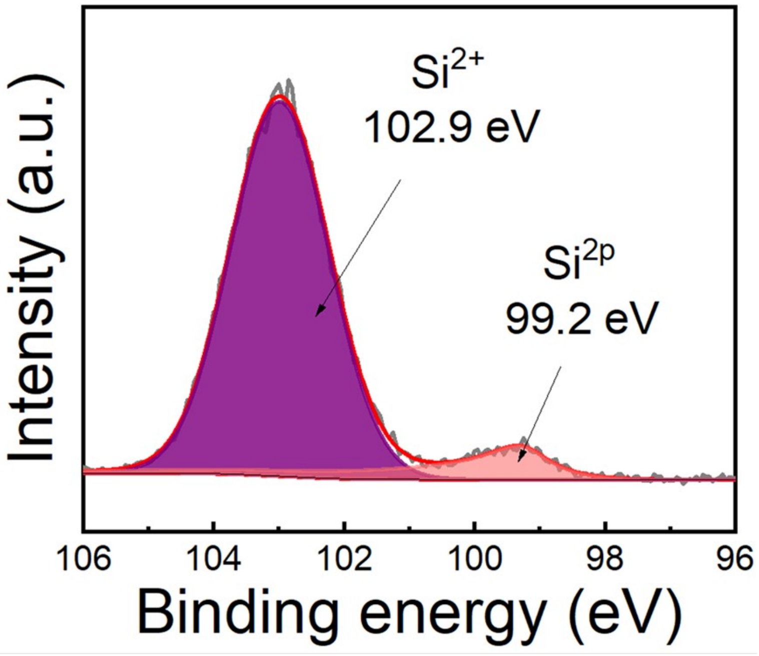

Fig S1: High-resolution XPS spectra of Si 2p.

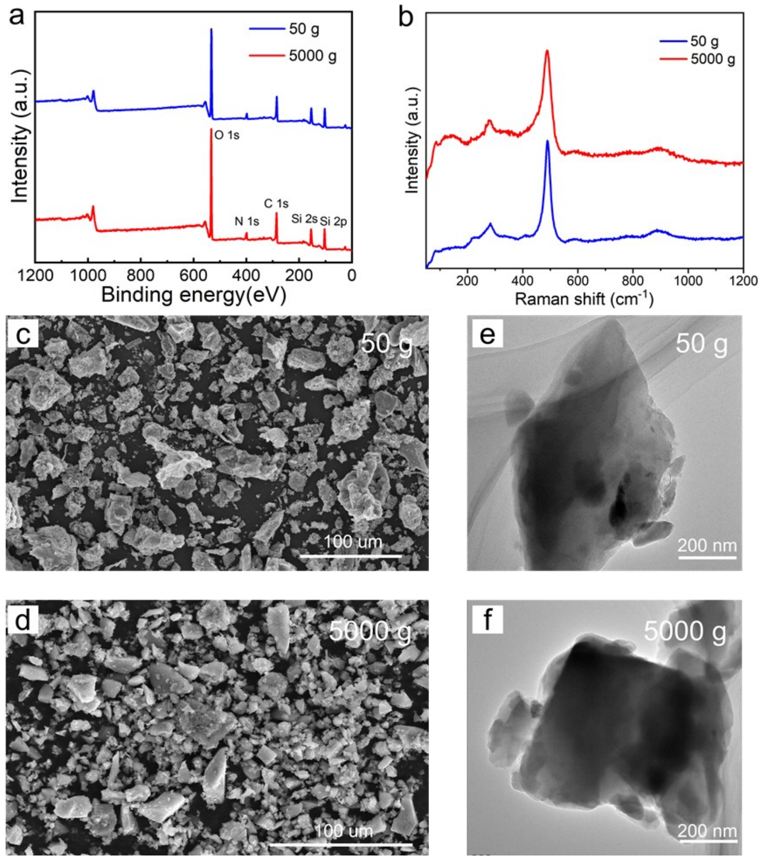

Fig S2. (a) X-ray photoelectron spectroscopy (XPS) full spectrum of 50g and 5000g CNS samples. (b) Raman spectrum of 50g and 5000g CNS samples. (c, d) scanning electron microscopy (SEM) image of 50g and 5000g CNS samples. (e, f) transmission electron microscopy (TEM) images of 50g and 5000g CNS samples.

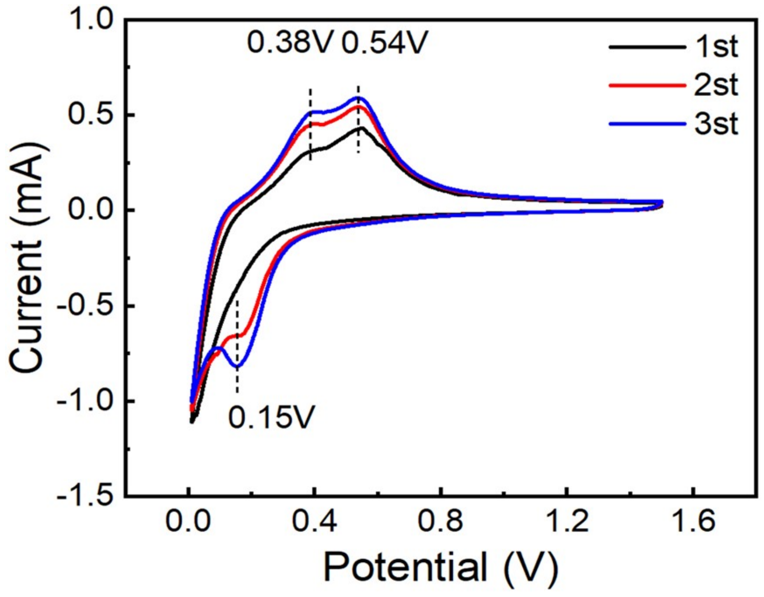

Fig S3: CV profiles of CS at 0.1 mV s^{-1}.

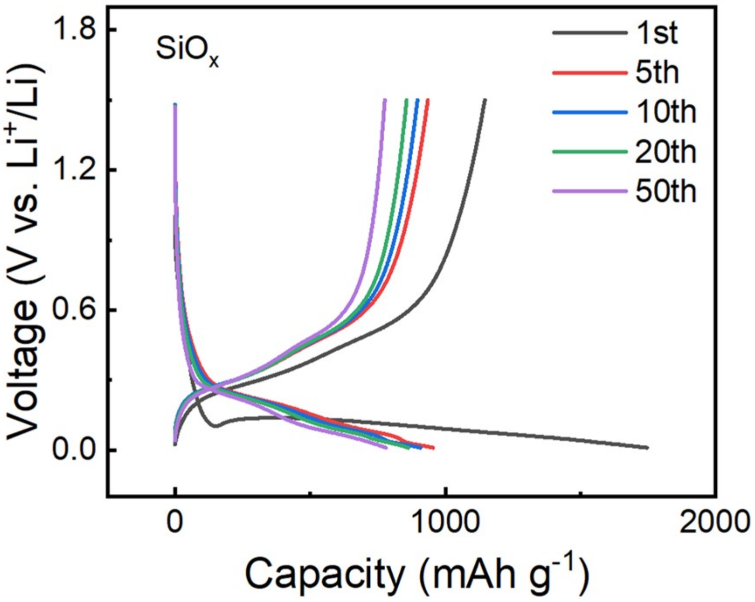

Fig S4: Discharge-charge voltage curves of SiO_x at 100 mAh g^{-1}.

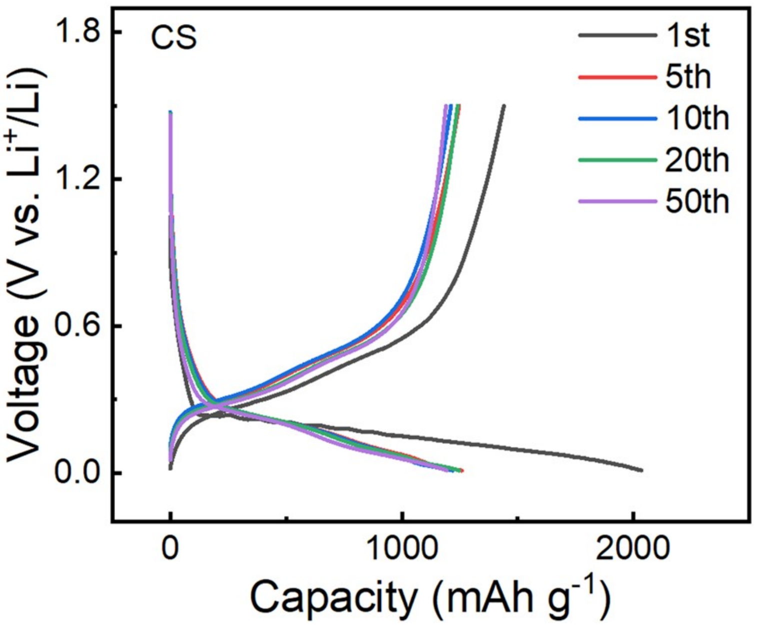

Fig S5: Discharge-charge voltage curves of CS at 100 mAh g^{-1}.

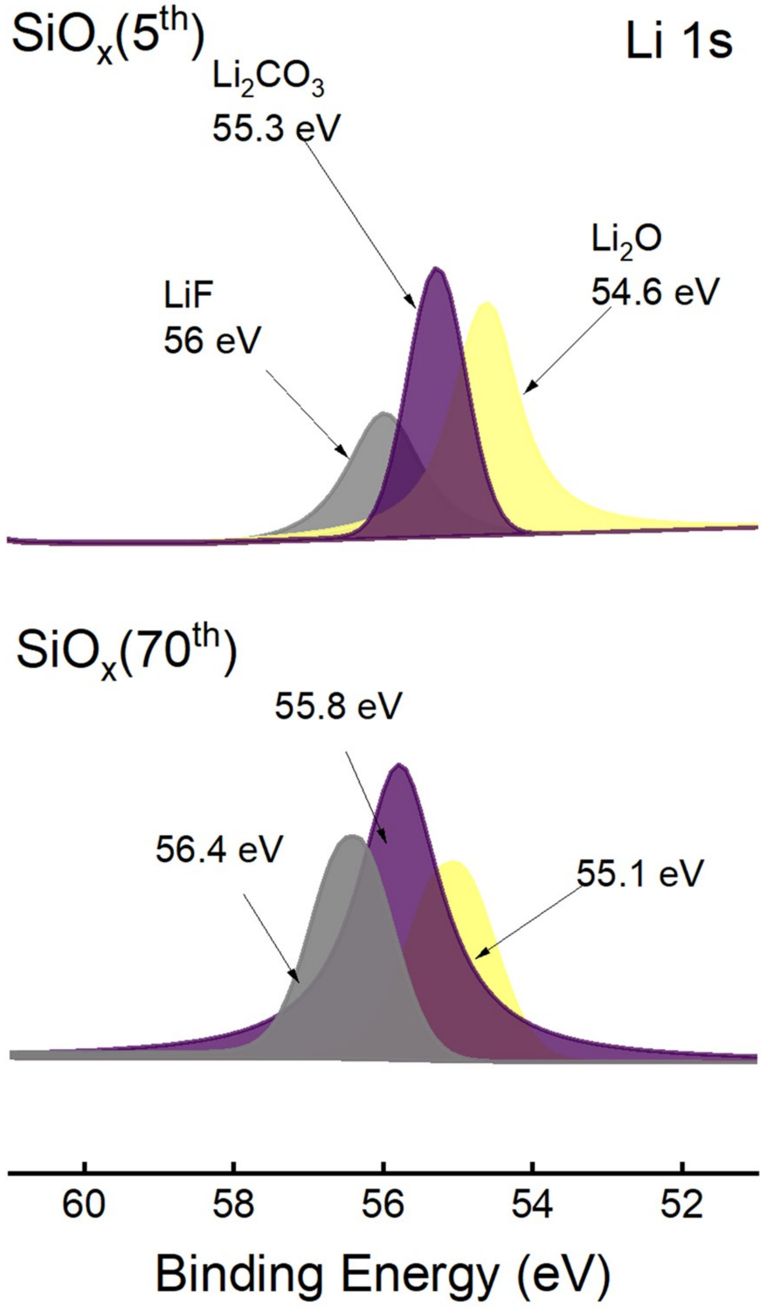

Fig S6: Comparison of high-resolution XPS spectra of Li 1s in the fifth and seventieth circles of SiO_x.

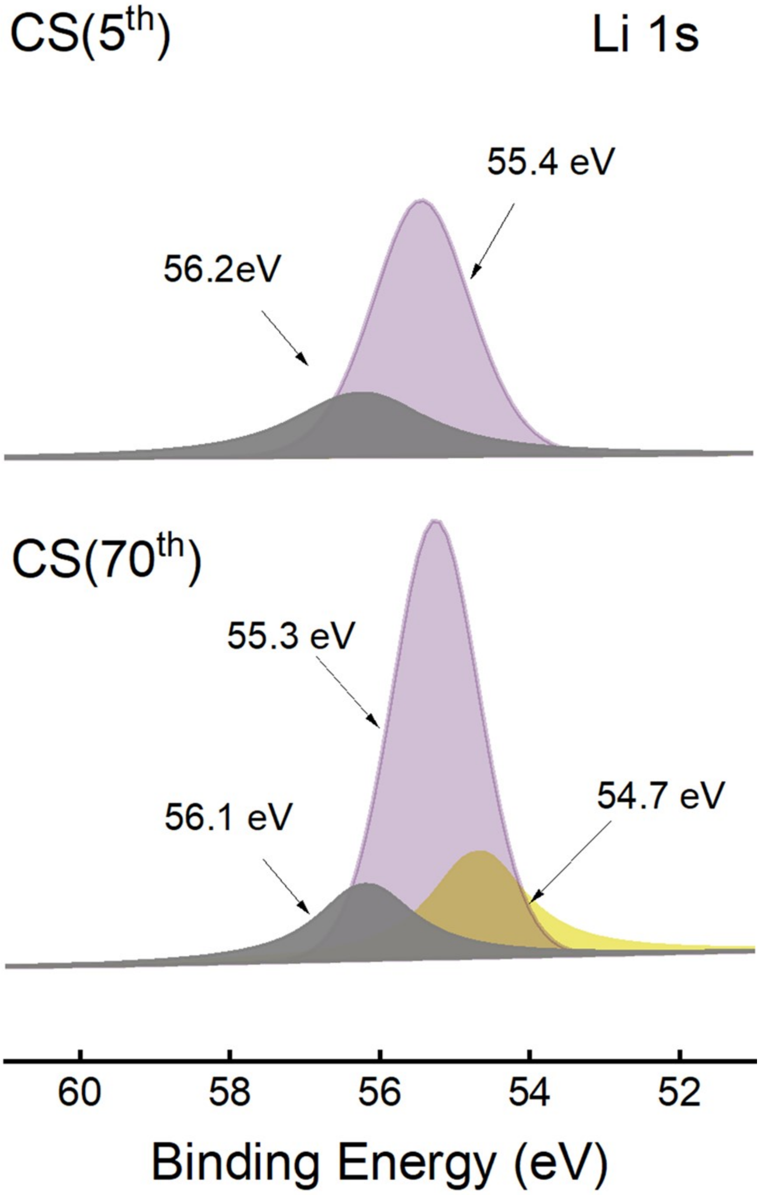

Fig S7: Comparison of high-resolution XPS spectra of Li 1s in the fifth and seventieth circles of CS.

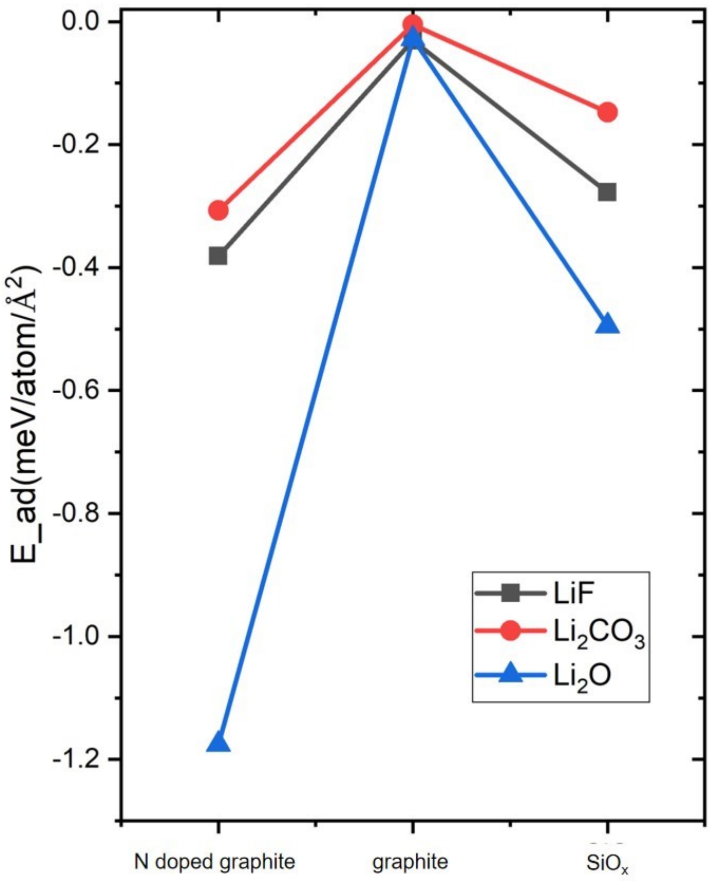

Fig S8: Formation of $LiF/Li_2CO_3/Li_2O$ adsorbed on the surfaces of N doped graphite, graphite and SiO_x, respectively.
